# Disseminated Mucormycosis with Characteristic Gastric Ulcers and Extensive Intravascular Invasion after ABO-Incompatible Living-Donor Liver Transplantation: A Case Report

**DOI:** 10.70352/scrj.cr.25-0651

**Published:** 2026-03-11

**Authors:** Satoshi Takada, Kento Terasaki, Ryosuke Gabata, Tomokazu Tokoro, Mitsuyoshi Okazaki, Yoshinori Takahashi, Yukinobu Ito, Akihiro Seki, Shinichi Nakanuma, Isamu Makino, Shintaro Yagi

**Affiliations:** 1 Department of Hepato-Biliary-Pancreatic Surgery and Transplantation, Kanazawa University, Kanazawa, Ishikawa, Japan; 2 Department of General Medicine and Infectious Diseases, JA Toyama Koseiren Takaoka Hospital, Takaoka, Toyama, Japan; 3 Department of Molecular and Cellular Pathology, Kanazawa University, Kanazawa, Ishikawa, Japan; 4 Department of Gastroenterology, Kanazawa University, Kanazawa, Ishikawa, Japan

**Keywords:** disseminated mucormycosis, zygomycosis, *Rhizopus microsporus*, gastric ulcer, intravascular lesion, ABO-incompatible living-donor liver transplantation

## Abstract

**INTRODUCTION:**

Mucormycosis is a life-threatening invasive fungal infection that occurs mainly in patients who are immunocompromised. Here, we report a case of mucormycosis with characteristic gastric ulcers and severe vascular invasion after ABO-incompatible living-donor liver transplantation.

**CASE PRESENTATION:**

A male patient in his 50s with decompensated cirrhosis due to metabolic dysfunction–associated steatohepatitis underwent an ABO-incompatible living-donor liver transplantation. Rituximab (375 mg/m^2^) was administered 14 days before transplantation, and tacrolimus (1 mg/day) and mycophenolate mofetil (500 mg/day) were initiated 7 days before surgery. Plasma exchange was performed the day before transplantation. Postoperatively, tacrolimus, mycophenolate mofetil, and methylprednisolone were continued as immunosuppressants, and micafungin was administered as antifungal prophylaxis. The postoperative course was complicated by sepsis due to infectious enteritis, which required broad-spectrum antibiotics. Two episodes of T cell-mediated rejection occurred, both treated with steroid pulse therapy. On POD 39, esophagogastroduodenoscopy was performed for persistent anemia and bloody drainage from the nasogastric tube. Multiple extensive gastric ulcers surrounded by dark brown mucosa were observed. Biopsy specimens revealed broad aseptate hyphae with wide-angle branching, consistent with mucormycosis. *Rhizopus microsporus*, the causative organism of mucormycosis, was isolated from gastric juice culture, confirming the diagnosis. Contrast-enhanced CT demonstrated poor enhancement of the gastric wall and multiple pulmonary nodules, suggesting disseminated fungal infection. Intravenous liposomal amphotericin B was initiated; however, on POD 56, the patient developed septic shock and died of multiple organ failure on POD 63. Autopsy revealed mucormycosis in the stomach, lungs, adrenal glands, thyroid gland, and kidneys. Cobblestone-like fungal masses were identified along the thoracic aortic intima, indicating extensive vascular invasion.

**CONCLUSIONS:**

This case represents a breakthrough mucormycosis infection that developed despite antifungal prophylaxis. The disease manifested as characteristic gastric ulcerations and extensive vascular invasion, leading to fatal outcomes despite liposomal amphotericin B therapy. Necrotic gastric ulcers and positive gastric juice cultures provide critical diagnostic clues. In highly immunosuppressed liver transplant recipients, atypical gastric findings should raise suspicion for mucormycosis, and early systemic evaluation is essential to improve otherwise poor prognoses.

## Abbreviations

EGD
: esophagogastroduodenoscopy
HEPA
: high-efficiency particulate air
L-AMB
: liposomal amphotericin B
LDLT
: living-donor liver transplantation
MMF
: mycophenolate mofetil
RAI
: rejection activity index

## INTRODUCTION

ABO-incompatible LDLT has been developed predominantly in Asian countries, where the shortage of deceased donors necessitates reliance on LDLT. Over the past two decades, the introduction of rituximab-based desensitization protocols and advances in perioperative management have markedly improved outcomes after ABO-incompatible LDLT. Nevertheless, the intensified immunosuppressive regimens required in this setting predispose recipients to severe infectious complications, which remain major causes of morbidity and mortality.

Mucormycosis is an uncommon but highly aggressive invasive fungal infection caused by organisms belonging to the order *Mucorales*. It typically occurs in profoundly immunocompromised hosts, including patients with hematological malignancies, diabetes mellitus, hematopoietic stem cell transplantation, or solid organ transplantation.^1)^ Although rhinocerebral and pulmonary involvement predominate, gastrointestinal mucormycosis is rare, accounting for fewer than 10% of cases. It carries a dismal prognosis, with a reported mortality rate of 40%–85%.^2)^ Early recognition is challenging because clinical manifestations are nonspecific and conventional diagnostic markers, such as serum β-D-glucan, are usually negative.

Only a few cases of mucormycosis have been reported in the context of LDLT, and gastrointestinal presentation is particularly rare. Here, we describe a fatal case of disseminated mucormycosis following ABO-incompatible LDLT, characterized by extensive gastric ulceration and remarkable intravascular invasion of the thoracic aorta. This report underscores the need for heightened clinical suspicion, especially in highly immunosuppressed transplant recipients with atypical gastrointestinal findings.

## CASE PRESENTATION

**Fig. 1:**
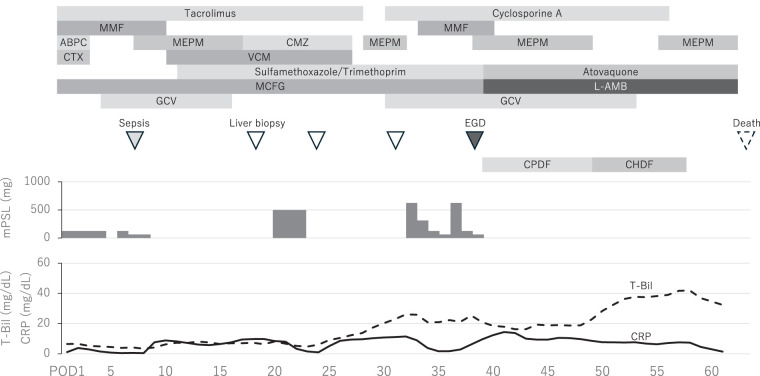
Clinical course after liver transplantation. The patient developed sepsis due to infectious enteritis, necessitating broad-spectrum antibiotics. Two episodes of T cell-mediated rejection occurred, both treated with steroid pulse therapy. Mucormycosis was identified by EGD on POD 39. Intravenous administration of liposomal amphotericin B was initiated; however, graft dysfunction worsened, leading to death from multiple-organ failure on POD 63. ABPC, ampicillin; CHDF, continuous hemodiafiltration; CMZ, cefmetazole; CPDF, continuous plasma filtration with dialysis; CRP, C-reactive protein; CTX, cefotaxime; EGD, esophagogastroduodenoscopy; GCV, ganciclovir; L-AMB, liposomal amphotericin B; MCFG, micafungin; MEPM, meropenem; MMF, mycophenolate mofetil; mPSL, methylprednisolone; T-Bil, total bilirubin; VCM, vancomycin

**Fig. 2:**
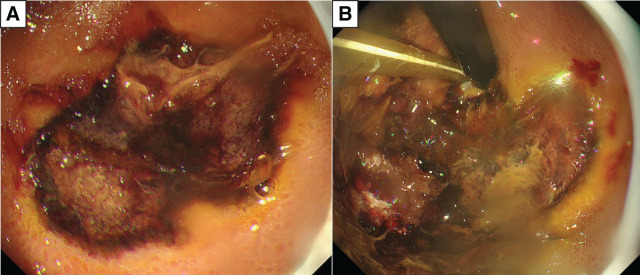
Esophagogastroduodenoscopy findings. (**A**) Irregular gastric ulcers with necrotic tissue–like deposits at their base. (**B**) Ulcer enlargement in the upper part of the stomach.

**Fig. 3:**
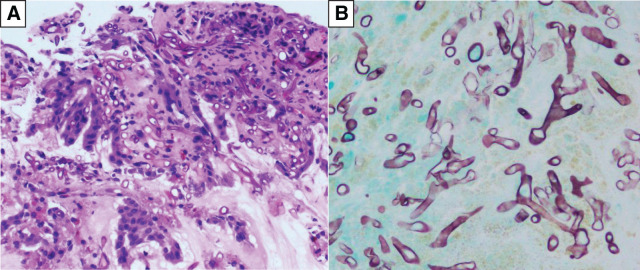
Pathological findings of gastric biopsy. Granulation tissue and mucosal epithelium are observed, within which broad aseptate hyphae with wide-angle branching were found on (**A**) hematoxylin and eosin staining and (**B**) Grocott’s methenamine silver staining.

**Fig. 4:**
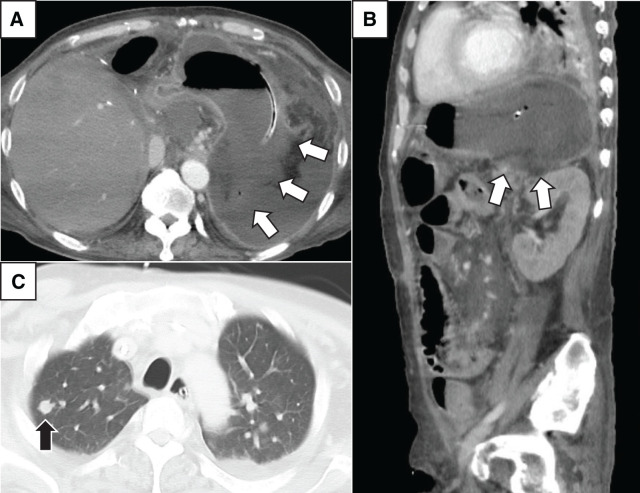
Contrast-enhanced CT findings. (**A** and **B**) White arrows show poor contrast enhancement of the gastric mucosa. (**C**) Black arrow indicates a pulmonary nodule, suggesting disseminated mucormycosis.

**Fig. 5:**
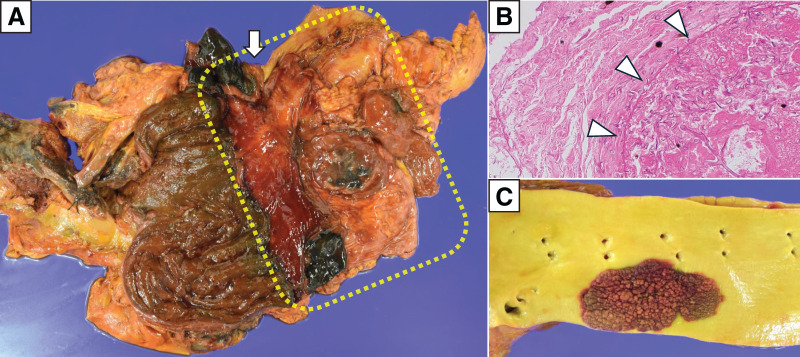
Pathological autopsy findings. (**A**) The area surrounded by the dotted line is the upper part of the stomach body, where the stomach wall had become necrotic and perforated. The white arrow indicates the esophagus. (**B**) Arrowheads show numerous fungal infiltrations into blood vessels on hematoxylin and eosin staining. (**C**) Cobblestone-like fungal masses were identified along the thoracic aortic intima.

A male patient in his 50s with decompensated cirrhosis due to metabolic dysfunction–associated steatohepatitis presented to our department for a liver transplant. He had been suffering from liver dysfunction for 8 years and was diagnosed with metabolic dysfunction–associated steatohepatitis by liver biopsy 6 years previously. At the time of consultation, his liver function was Child-Pugh grade C (13 points), and the Model for End-Stage Liver Disease score was 18 points. The patient presented with esophageal and rectal varices, splenomegaly, and portal hypertension. He had a history of long-term antibiotic therapy for colitis-associated sepsis and trauma-associated methicillin-susceptible *Staphylococcus aureus* bacteremia within 6 months prior to transplantation. The patient had recurrent hepatic encephalopathy and progressive sarcopenia, making it difficult to wait for a deceased donor liver transplant; therefore, early liver transplantation was considered desirable. The patient underwent ABO-incompatible LDLT using a right lobe graft from his 48-year-old spouse (donor blood type: B, recipient blood type: O), along with a splenectomy, as this case meets the risk criteria for early graft failure.^3)^ Rituximab (375 mg/m^2^) was administered 14 days before transplantation to delete B cells, according to Japanese guidelines. Tacrolimus (1 mg/day) and MMF (500 mg/day) were administered 7 days prior to transplantation. In addition, plasma exchange was performed to decrease anti-B antibody titer the day before transplantation. The patient underwent routine ear, nose, and throat evaluation prior to transplantation, and no sinusitis or fungal lesions were detected. Tacrolimus, MMF, and methylprednisolone were administered postoperatively. Intravenous micafungin was administered to prevent fungal infections. The postoperative course is summarized in **Fig. 1**. On POD 8, the patient was diagnosed with infectious enteritis and sepsis due to extended-spectrum beta-lactamase–producing *Escherichia coli* and was treated with meropenem. On POD 19, reduced portal vein blood flow accompanied by elevated inflammatory markers prompted a liver biopsy, which suggested T cell-mediated rejection with an RAI of 2. Steroid pulse therapy with methylprednisolone (10 mg/kg = 500 mg/body) was initiated. Owing to concerns regarding susceptibility to infection, the therapy was discontinued after 3 days; nevertheless, portal vein blood flow and inflammatory markers subsequently improved. On POD 31, portal vein blood flow again decreased, and repeat liver biopsy demonstrated T cell-mediated rejection with an RAI of 5. Accordingly, steroid pulse therapy was repeated. Platelet counts remained low, and tacrolimus-associated thrombotic microangiopathy was also considered a differential diagnosis; therefore, tacrolimus was changed to cyclosporine A. On the same day, cytomegalovirus monitoring demonstrated positive results, and ganciclovir was initiated. During the same period, anemia and bloody drainage from the nasogastric tube persisted. On POD 39, EGD revealed multiple extensive ulcers surrounded by dark brown mucosa extending from the gastroesophageal junction to the upper stomach (**Fig. 2**). Hematoxylin-eosin and Grocott’s methenamine silver staining of the ulcer specimen showed broad aseptate hyphae with wide-angle branching (**Fig. 3**). Immunostaining for cytomegalovirus revealed negative results. Contrast-enhanced CT revealed poor contrast in the stomach wall (**Fig. 4A** and **4B**) and multiple lung nodules (**Fig. 4C**). Serum β-D-glucan and blood cultures were negative. Mucormycosis was diagnosed based on the morphological characteristics of the mycelia. Later, *Rhizopus microsporus*, one of the causative organisms of mucormycosis, was detected in gastric juice cultures, confirming the diagnosis. At that time, the patient suffered from graft failure and severe myelosuppression; multiple pulmonary nodules were considered disseminated lesions of mucormycosis, and surgical resection of the infected lesions was not considered feasible. L-AMB (5 mg/kg) was administered intravenously to treat disseminated mucormycosis. However, on POD 56, the patient developed septic shock, and contrast-enhanced CT showed worsening contrast enhancement of the gastric wall and enlargement of the lung nodules. On POD 63, the patient died of multiple-organ failure. Autopsy revealed mucormycotic lesions in the stomach, lungs, adrenal glands, thyroid gland, kidneys, and lower thoracic aortic intima (**Fig. 5**).

## DISCUSSION

This case highlights that echinocandin-based prophylaxis does not prevent mucormycosis and that clinicians should maintain a high index of suspicion for breakthrough infections in severely immunosuppressed liver transplant recipients. Echinocandins have no activity against *Mucorales* and therefore cannot prevent mucormycosis. According to the 2019 consensus definitions of the Mycoses Study Group Education and Research Consortium and the European Confederation of Medical Mycology (MSG-ERC/ECMM),^4)^ a breakthrough invasive fungal infection is defined as any invasive fungal infection that occurs during exposure to an antifungal agent, including infections caused by fungi outside the spectrum of activity of the administered drug. Breakthrough invasive fungal infections during echinocandin therapy are increasingly recognized, particularly in hematology and transplant populations.

Beyond its occurrence as a breakthrough infection, the patient’s clinical course illustrates the fulminant nature of gastrointestinal mucormycosis. The gastrointestinal tract is rarely the primary site of mucormycosis, with an estimated incidence of approximately 7%. Gastrointestinal mucormycosis carries an extremely poor prognosis, with a reported mortality rate of up to 85%. Dissemination to non-contiguous organs occurs in nearly 40% of cases and is often fatal.^5)^ Clinically, gastrointestinal mucormycosis presents with nonspecific symptoms such as abdominal pain, fever, and vomiting, and frequently causes gastrointestinal bleeding and perforation.^2,6,7)^ Consistent with the present case, gastric involvement is typically characterized by ulcerative lesions with extensive necrotic tissue.^8,9)^

Although the vascular lesions observed in this case were atypical, they clearly demonstrated the marked angioinvasive nature of mucormycosis.^5,10)^ This disease has a strong tendency to cause vascular thrombosis and tissue necrosis, which likely result in impaired delivery of antifungal agents to infected sites. Given these pathological characteristics, early and aggressive surgical resection should be considered whenever feasible.

L-AMB is the first-line drug for the treatment of mucormycosis, and posaconazole or isavuconazole may be used as second-line drugs or in combination.^11)^ International guidelines recommend the use of amphotericin B at a dose of 5–10 mg/kg.^11)^ However, a high rate of reversible renal dysfunction has been reported at a dose of 10 mg/kg^12)^; therefore, caution should be exercised. In this case, the patient was started on 5 mg/kg/d, and the disease seemed to have temporarily settled, but the patient’s condition deteriorated rapidly on POD 56, and the decision was made not to aggressively treat the patient. Considering the patient’s poor general condition and the fact that the disease was already disseminated at the time of diagnosis, increasing the dosage of amphotericin B or combining it with posaconazole or isavuconazole should have been considered.

One of the main reasons for the poor prognosis of mucormycosis is the difficulty of early diagnosis, as reliable imaging findings or serum biomarkers have not yet been established. Serum β-D-glucan levels and blood cultures are usually negative^13)^; therefore, the diagnosis relies primarily on histopathological examination, tissue culture, and PCR-based methods.^14)^ As a result, mucormycosis is sometimes diagnosed only at autopsy.^1)^ In the present case, the diagnosis was established based on characteristic ulcerative gastric lesions, with confirmation by histopathological examination and culture of gastric drainage fluid. Although comprehensive studies evaluating the diagnostic utility of gastric juice cultures are lacking, previous reports have described successful isolation of causative fungi from mucus obtained from ulcerative gastric lesions.^9)^ These findings suggest that gastric fluid culture may represent a useful adjunct for the diagnosis of gastric mucormycosis.

We searched PubMed for reports of mucormycosis in LDLT and found only 4 cases^8,15–17)^ (**Table 1**). The reported cases were from India and Japan, all of which involved men. Only 1 case of ABO incompatibility has been reported in Japan. Only 1 patient showed involvement of the gastrointestinal tract, which was diagnosed by histopathological examination after resection for intestinal perforation, and that patient survived. All patients were diagnosed based on histopathological examination or lesion cultures. Three patients, including the present case, died in the early postoperative period despite treatment with L-AMB.

**Table 1:** Reported cases of mucormycosis after living-donor liver transplantation

Authors	Year	Age	Sex	Country	ABO Compatibility	Immunosuppressant	Primary infection site	Diagnostic process	Medication	Surgery	Outcome
Lalwani^8)^	2012	9 m.o.	Male	India	Matched	Tacrolimus, MMF	Jejunum	Perforation → Jejunectomy → Pathological exam	L-AMB (7 mg/kg)	Jejunectomy	Survived
Sethi^15)^	2016	44 y.o.	Male	India	Matched	Basiliximab, Tacrolimus, MMF	Graft	Hepatic hypoechoic mass → Culture of aspiration specimen	L-AMB → Itraconazole, Caspofungin, Posaconazole	None	Died (3 weeks after LDLT)
Chaundhary^17)^	2020	57 y.o.	Male	India	Matched	Tacrolimus, MMF, Steroid	Graft	Hepatic dysfunction → Liver biopsy	L-AMB (5 mg/kg), Posaconazole	None	Survived
Mita^16)^	2022	47 y.o.	Male	Japan	Incompatible	Rituximab, Tacrolimus, MMF, Steroid	Lung	Sputum culture	L-AMB (5 mg/kg)	Partial lung resection	Died (4 weeks after LDLT)
Our case	2026	56 y.o.	Male	Japan	Incompatible	Rituximab, Tacrolimus, MMF, Steroid	Stomach, Lung	Gastric ulcer →Biopsy and culture of gastric fluid	L-AMB (5 mg/kg)	None	Died (9 weeks after LDLT)

L-AMB, liposomal amphotericin B; LDLT, living-donor liver transplantation; MMF, mycophenolate mofet

Finally, we investigated the route of fungal infection in this patient but were unable to identify the exact source of infection. After transplantation, the patient was managed in the ICU. In our institution, the ICU is air-conditioned with HEPA filtration; however, strict positive-pressure isolation is not uniformly implemented in each room. The patient had no relevant social, occupational, or travel exposures, and no hospital construction or renovation occurred during the hospitalization. While the exact source could not be determined, airborne inhalation remains the most plausible route given the profound immunosuppression.

## CONCLUSIONS

Collectively, our case underscores the need for heightened clinical suspicion of mucormycosis in highly immunosuppressed transplant recipients presenting with atypical gastrointestinal lesions. Characteristic gastric ulcers and positive gastric juice cultures may provide important diagnostic clues, enabling earlier initiation of therapy. Nevertheless, outcomes remain poor in disseminated disease, and further studies are required to establish strategies for early detection and effective management in liver transplantation.

## DECLARATIONS

### Availability of data and materials

The datasets used and/or analyzed in the current study are available from the corresponding author upon reasonable request.
